# Development and evaluation of a school-based physical literacy intervention for children in Germany: protocol of the PLACE study

**DOI:** 10.3389/fspor.2023.1155363

**Published:** 2023-05-31

**Authors:** Johannes Carl, Louisa Schmittwilken, Katharina Pöppel

**Affiliations:** ^1^ Department of Sport Science and Sport, Friedrich-Alexander University Erlangen-Nürnberg, Erlangen, Germany; ^2^ Institute of Sport Science, Oldenburg University, Oldenburg, Germany

**Keywords:** active lifestyle, education, enrichment, exercise, health, physical activity, practice, program

## Abstract

**Introduction:**

Fueled by the COVID-19 pandemic, the physical activity behavior of children has reached a concerning level nowadays. By empowering individuals to be physically active throughout the life course, the concept of physical literacy has recently gained increasing attention and adopts a holistic-integrative perspective on PA promotion. Although the field has successively attempted to translate the conceptual ideas of physical literacy into interventions, the theoretical base is heterogeneous and is often lacking within interventions. Furthermore, several countries, including Germany, have not equally adopted the concept yet. Therefore, the goal of the present study protocol is to describe the development and evaluation approach of a PL intervention (“PLACE”) for children in grades three and four within the German all-day schooling system.

**Methods:**

The physical literacy intervention cultivates explicit theory-content links and comprises 12 heterogeneous sessions (each 60-90 min in length). The study contains three different phases with two initial pilot studies and a subsequent main study. The two pilot studies take a mixed-methods character by drawing on quantitative pre-post-designs as well as interviews with children (in groups). In the main study, we will longitudinally compare the course of PL values (five outcome domains: physical, affective, cognitive, social, behavioral) between two study arms: school classes of children are either assigned to an intervention condition (regular physical education and health care plus PL intervention) or to a control condition (regular physical education and health care only).

**Discussion:**

The findings of this study will provide evidence on how to structure a multicomponent intervention in Germany based on the PL concept. In summary, the results will report on the effectiveness of the intervention and, therefore, decide whether to scale-up the intervention.

## 1. Introduction

### 1.1. Children's physical activity behavior in the post-COVID time

The prevalence of physical inactivity has reached a considerable level across the globe (1, 2) and has, therefore, culminated in specific recommendations and guidelines to counteract such negative developments (3). However, there is sufficient evidence underlining that the COVID-19 pandemic and its concomitant mobility restrictions, which have affected physical activity (PA) services over weeks and months, have even intensified this situation (4–6). When identifying a target group that has undergone particular reductions in physically active lifestyles in this phase, on the one hand (7), and who are in a crucial phase for motor development on the other (8), children come to the focus of interest. In general, experts suggest individuals under the age of 12 years to exert at least 60 min of moderate-to-vigorous PA per day (9). However, the majority of children and adolescents on the globe (81.0%) and in Germany (83.4%) did not meet these recommendations even before the pandemic (1). According to a longitudinal study in Germany, sport activities of childen aged 4–17 have decreased from 32.5 min per day before the pandemic to 23.9 min per day during the first lockdown and to 13.6 min per day during the second lockdown (7). Simultaneously, children reported lower health-related quality of life, more mental health problems (17.8%), and higher anxiety levels (24.1%) than before the pandemic (10). Unfortunately, there is sparse data on how PA patterns have changed after the first two years of the pandemic. However, it can be questioned whether the PA behavior in children will fully return to the level before 2020. From a public health perspective, the adoption or stabilization of inactive habits warrants concern, as longitudinal studies have highlighted that PA patterns often track into adulthood (11, 12). Hence, it is likely that this negative PA trend in the society negatively influences individual's health in the long term (13). In summary, strong arguments are given to specifically promote physically active lifestyles among children (14, 15).

In addition to the mere quantity of PA, children also did not gain the necessary qualitative experiences during the main phases of the pandemic. Home schooling and mobility restrictions have, to a substantial degree, prevented sport clubs or teachers (in physical education) from conveying important qualifications for physically active lifestyles (16, 17). Actually, actors in these important settings basically have the potential to build proficient motor skills, create enjoyable atmospheres of PA, provide valuable social interactions, and stimulate reflections related to PA (18–20). However, even if the pandemic situation permits to arrange sport or physical education practices as usual, stakeholders typically prioritize physical and psychomotor input over cognitive and affective experiences (21). Arising from this overall constellation, PA promotion strategies should be installed that elicit the full range of positive experiences among children simultaneously (22).

### 1.2. Physical literacy

Given this claim, a theoretical concept is required that holistically encompasses the multifaceted individual experiences in the context of physically active lifestyles. The concept of physical literacy (PL) has gained increasing attention in recent years (23, 24) and has found entrance into important documents related to PA, sport, and physical education, including the Global Action Plan on Physical Activity 2018–2030 (15) and the UNESCO Quality Physical Education Guidelines for Policymakers (25). Many studies and theoretical reports (23, 26) refer to the PL definition of the International Physical Literacy Association (IPLA) which comprehends PL as “the motivation, confidence, physical competence, knowledge and understanding to value and take responsibility for engagement in physical activities for life” (27). Sport Australia describes a physically literate person as someone who draw on his/her “integrated physical, psychological, social and cognitive capabilities to support health promoting and fulfilling movement and physical activity—relative to their situation and context—throughout the lifespan” (28). The aspect of *integration* within this definition harmonizes with assumptions of monism or embodiment as frequently expounded philosophic underpinnings of PL emphasizing that body and mind act as inseparable units of human existence (22, 29). Albeit sharing certain principles and core domains (physical, affective, cognitive), there is no global consensus about the core of PL (26, 30, 31). Instead, the PL field can be characterized through different conceptualizations and, accordingly, different networks “defending” their own understanding of PL (30, 32). Nonetheless, the understanding PL is highly embedded into the cultures and traditions of the corresponding countries (23, 33, 34).

Not all countries have yet elaborated their own understanding of PL (26, 35). One of these countries is Germany, where “Bildung” (36, 37) or “competencies” (38–42) are discussed more strongly within the field of person-related health promotion and where curricula for education (including physical education) should be aligned with the idea of “competencies” (43–45). Accordingly, a deficit of, but also an added value of research activities in the field of PL has been identified for Germany (34, 46). Due to this lack, it is necessary to explicate the theoretical lens that is specifically adopted by a PL study (31). In this regard, the present endeavor draws on a PL working definition that comprises individuals' physical, cognitive, psychological, and social requirements for PA behavior (47). With this definition, the current study favors the consideration of a social aspect—an aspect that is in the focus of current discussions and emphasized through its introduction by the Australian PL framework (47). As a consequence of adhering to this conceptualization, PL interventions should deliberately target the physical, cognitive, psychological, and social domains in consideration of the Australian framework. This claim can be met by cultivating explicit links between theoretical components and the interventional content (40). According to a recent review, PL interventions insufficiently account for the holistic character of the theoretical approach, as the cognitive and especially the affective domains are often neglected in PL interventions (24). Despite this deficit on the theoretical level, PL interventions have the potential to entail significant effects on the PL dimensions when integrating corresponding operationalizations for the different domains (also including PA levels) (48). Supported by empirical evidence that PA interventions achieve larger effects if they possess sound theoretical foundations (49), initiatives to promote PL should attempt to be based on theory and avoid substantial “uncouplings” from the original PL concept (50). This demand can be realized by providing a tight interlocking with PL theory throughout the entire process of intervention development (24).

### 1.3. Research questions, goals, and hypotheses

The main goal of the present study is to investigate the effectiveness of a PL intervention in different schools in the city and city state of Bremen, Germany. This study addresses the following primary research question: *Can an intervention with clear foundations in the integrated PL domains systematically promote PL among children 8–11 years of age?* Accordingly, we will test the main hypothesis (one-sided procedure) that children in the intervention condition develop significantly better over the interventional period in PL outcomes compared to children in the control condition.

In addition, the present study pursues two sub-goals on its way toward the primary goal: (a) to derive holistic, age-specific intervention components for 8–11 years old children with explicit manifestations in the PL concept, and (b) to implement the intervention in cooperation with primary schools in the city and city state of Bremen, Germany. In case of a successful implementation and evaluation (i.e., approval of the research question), the present study also reserves capacities for potentially preparing the dissemination of the intervention concept.

## 2. Methods

### 2.1. Study design

**Figure 1:**
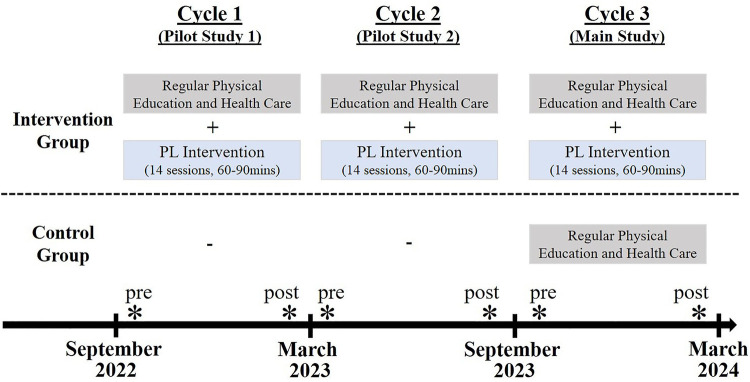
Study design.

For the stepwise development, advancement, and evaluation of the intervention concept, we will employ a combination and series of different studies with ascending methodological rigor (51). Given that clear orientations and examples of interventions based on PL are missing for Germany (e.g., components for practices) (46), the present study will make conceptual and interventional groundwork. Therefore, we will start with two pilot studies in two consecutive cycles (see Figure 1). In these two cycles, we will derive a intervention (goal a) based on central literature for the practical delivery of PL (24, 47, 52, 53), and implement the theoretically derived content for the first time in practice (goal b). Formative and process evaluations will allow to revise and further advance the PL intervention on the grounds of feasibility and practicability arguments, on the one hand, and empirical arguments, on the other. The pilot studies will be performed between September 2022 and February 2023 (cycle 1) and between March 2023 and August 2023 (cycle 2), respectively, in a non-control group design with two measurement times (see Figure 1). From a quantitative perspective, we will carry out pre-post analyses with the PL outcomes. From a qualitative perspective, we will perform group interviews with participating children (54, 55) by taking a retrospective view on the PL concept and intervention content. The delivery of the intervention will be accompanied by a multiperspective panel that monitors the delivery and facilitates additional adjustments through transdisciplinary discussion (56).

After the completion of the two pilot studies, we will follow up with the main study. The main study will more rigorously examine the effectiveness of the final PL intervention in a controlled design. In each school, an even number of classes of one grade level will participate in the study. Half of the classes will be assigned to the intervention condition, which will pass regular half-year education in primary school plus a weekly PL intervention. In contrast, the other half of the classes will be assigned to the control condition, which will undergo only regular half-year education in primary school without the additional PL intervention (see Figure 1). Randomization cannot be fully realized as pragmatic reasons co-influence the assignment to the different intervention arms (57). The main study, which also includes two measurement time points (e.g., pre and post), will take place between September 2023 and February 2024. In summary, the research design with two pilot cycles (51) accounts for potential pitfalls (e.g., revision of PL content) toward a sound examination of the effectiveness of the PL intervention. The final reporting of study will adhere to the recent physical literacy interventions reporting template suggesting a total of 14 items (58). The ethics committee of the University of Oldenburg, Germany, has approved the conduction of the study (sign Drs.EK/2022/057).

### 2.2. Setting, participants, and recruitment

PL can be developed in and across different contexts and environments (31, 59, 60). In the light of the existing compulsory attendance, school activities have the advantage that they reclaim to reach almost all children, regardless of the socioeconomic status or other heterogenous characteristics (61). Therefore, schools constitute a promising setting for health-promoting initiatives, in general, as well as PA initiatives, in specific (61–63). However, in contrast to Canada (64), Australia (65) or the United States (66), PL is not a goal within physical education curricula in Germany (46). To create a person-centered atmosphere without pressure regarding grades, our research team placed strong emphasis on decoupling the intervention from the regular schedules of physical education (67) by exclusively focusing on extracurricular time (“außerunterrichtlicher Bereich”) at schools.

The present study will take place within the all-day schooling system of primary schools in the city state of Bremen, Germany. Prior to the study, the research team contacted the school/education authorities of the city state of Bremen, which coordinate and administer extracurricular programs for children. The authorities provided a list of schools that the research team was entitled to contact within the scope of a program for children mitigating health-related consequences after the COVID-19 pandemic. In this context, the research team successively contacted the school administrators (and in single cases also sometimes the corresponding discipline leaders) of the primary schools and invited them for participation in the study. All PL activities as additional (educational) enrichment for children will take place outside the regular morning lessons to ensure that no regular physical education classes are replaced and to identify optimal slots within the weekly schedules. For the organization, the research team will closely communicate with the schools (53, 68) to find school- and class-specific solutions (e.g., slots in gymnastic halls, free times, availability of activity material).

The focus of the PL interventions will be placed on pupils of the third or fourth grade, wherein the class will serve as the level for the assignment to study conditions. Accordingly, the present study will address children aged between 8 and 11 years within the trial and analyses. Furthermore, children and their legal guardians will have to provide informed consent to study participation. In line with the inclusive potential of PL (69), children with diagnosed special needs will be invited to join the intervention (as long as they can participate in regular physical education) and evaluation. Nevertheless, they will be excluded from the analysis for psychometric reasons.

### 2.3. Intervention

#### 2.3.1. Intervention structure, organization, and content

To develop a holistic, age-specific intervention for 8–11 years old children theoretically based on the PL concept (goal a), we will deliver an intervention program comprising a total of 14 individual sessions. The duration of the sessions will be aligned with the logistic and temporal situation at the recruited schools and can range between 60 and 90 min. To facilitate the arrangement of the school schedules and promote comparability, the frequency of sessions held will be one time per week. Each session will be driven by the concept of PL and will be implemented after the regular classes, e.g., in times of afternoon care, or in some schools in the context of an individual spare time in between the regular school day. In contrast to theory-inspired interventions, theory-based interventions cultivate explicit links between theory and content (70, 71). In the context of this intervention, each session will contain explicit links to all PL domains (see Table 1) in accordance with the selected definition and framework of PL (47): the physical, the cognitive, the affective, and the social domain. All sessions will be realized via actual movement (PA behavior), which implicates that theoretical inputs without an immediate transfer into practice will be avoided across the sessions. In reference to studies which criticized the separation of the different domains in previous PL interventions or the emphasis on a specific domain (the physical domain foremost) (24, 72), our aim will be to consider the integrated nature of PL in every single session. Accordingly, we will not treat the PL domains as isolated blocks, instead addressed the domains in an integrative manner (73).

**Table 1:** A detailed overview of the theory-based PL intervention content.

Session	Main focus	Main goal	Theory-content link			
			Physical domain	Cognitive domain	Psychological (affective) domain	Social domain
1	Evaluation: Pretest	Self-evaluation, enabling PL charting	Mixed rule-based games focusing object control	Content knowledge: Reasons for evaluation	Self-perception	Fairness, inclusion
2	Cooperative game forms	Strengthen group structure	Game arrangements promoting cooperation and anticipation	Strategy and planning; Participatory development and operation with cooperation strategies	Focusing success experiences with the whole group	Communication; respect; solve team challenges
3	Ball games (Part 1)	Improve object control	Promoting object control (ball), throw, catch, shoot within different individual exercises, and team games	Knowledge about central skills for ball manipulation	Individual progress and confidence in object control	Playing in different ways together and against each other
4	Acrobatics	Experience different acrobatic formations	Individual, pair, and group arrangements focusing on static strength and promoting group balance	Building up a repertoire of basic acrobatic forms; characterizing “physical activity”	Focus on courage, self-awareness, and trust (e.g., in building a pyramid)	Promoting communication, collaboration, and integrity
5	Scuffling	Regulate one's strength	Station run with different tasks (e.g., push, pull, hold) fostering strength and stability	Internalizing rules for scuffling against someone	Self-regulation and proper usage of own strength	Respect towards others, their body, and limits
6	Endurance games	Pacing one's energy sources	Game arrangements challenging individual endurance boundaries	Understanding the pulse, observing its reaction to a sport activity	Volition to maintain the load throughout an entire game; fostering perseverance	Support and cheer up others, relationships
7	Racket sport	Get in touch with different types of racket sport	Exercises focusing basic racket handling (e.g., via hockey, badminton, or tennis rackets), introduction in small games using the rackets	Reasoning: Advantages of (regular) PA	Confidence in handling equipment	Playing in different ways together and against each other
8	Parkour	Development and proper use of movements in the context of parkour	Basics of parkour; jumping, running, and overcoming obstacles; agility exercises	Knowledge about parkour as a lifestyle activity in urban spaces; linking obstacle equipment from indoor use to self-initiated outdoor use	Self-awareness of skills, overcoming obstacles	Respect toward others and their abilities, integrity
9	Dancing	Become acquainted with aesthetic and rhythmic movements	Rhythmic movements, perform aesthetic movements in a group	Knowledge about the variety of dancing; developing dances in accordance with the rhythm	Enjoyment; self-expression through dancing	Development of a group choreography (for communication and collaboration)
10	Ball games (Part 2)	Improve object control	Object control (ball); Exercises focusing the reaction time	Knowledge about different types of coordination	Celebrate progress (Ballgames-1)	Playing in different ways together and against each other
11	Trend sport	Learn something new	Object manipulation: throwing and catching a frisbee	Reasoning: issues of being active every day—discuss solutions	Encouragement and motivation to try something new	Trying something new together, society and culture
12	Conditional abilities	Introduction in different conditional abilities, own strengths	Small team games focusing the range of movements running, throwing, pushing	Introduction in knowledge of different abilities to be physical active	Enjoyment of various movements; focusing individual success experiences	Respect individuality; communication, ethics
13	Free session	Enjoyment; implement own movement ideas	Mixed games and exercises	Reflection of “taking home” messages of the program	Involvement; encouragement	Communication; relationships
14	Evaluation: Posttest	Self-evaluation, promoting PL charting	Mixed rule-based games focusing object control	Changes in PA/sport habits before and after	Self-perception	Respect towards others, fairness

The intervention will be designed as a 14 week-program, while evaluations (pre and post) will be integrated into the first and last session. In addition to the scientific purpose, the evaluation will promote individuals' charting of PL, thus enabling reflections about one's journey and progress (74). Irrespective of these evaluation sessions, each session will contain a specific topic. In line with Whitehead (75) who emphasized the benefit of offering a wide range of contents to enhance the motivation of children to become physically active, we will cover a broad range of physical activities across the entire program, as PL (with its holistic and monistic view) advocates for engaging in encompassing physical activities and for making experiences with their own physical capacities (22, 60). We will consider rule-based games, aesthetic movements, movements with and on equipment, racket sports, scuffling, and basic forms of fitness (76). More specifically, we will realize rule-based games primarily via ball games and racket sports; the aesthetic input will focus on dancing and acrobatics, and fitness will be dominantly targeted via endurance-oriented games or in the context of parkour (for details, see Table 1). Furthermore, differentiations (taxonomies) between locomotion vs. object control (77) as well as individual vs. team activities (78) will guide the intervention to cover the breadth of movement forms and experiences. We will concentrate on land-based experiences, as aquatic activities cannot be realized for logistic-pragmatic and legal-qualificatory reasons.

When explicating the integrated mediation of the PL domains, the physical domain will be interventionally connected to the cognitive domain through theory-based inputs in each session. In line with Keegan et al. distinguishing six elements for the cognitive domain (i.e., content knowledge, rules, strategies and planning, tactics, awareness, as well as purposing and reasoning) (45), we will cover these different elements in specific session. Following this, we will define rules for scuffling, exchange strategies, and plans in the context of cooperative game forms or discuss tactics referring to various game arrangements. Furthermore, we will integrate reflections and phases to exchange knowledge about skills for ball manipulation as well as discuss purposes and reasons for being active on a regular level (for details, see Table 1). Finally, for the cognitive domain, we will verbalize previous and upcoming movements as well as reflect movements of other children (79). Short theory-based inputs will be included as transitions between games, little breaks, or during some games (e.g., modifying tactics).

The affective domain will be conveyed in each session by transferring and supporting principles of motivation, autonomy, enjoyment, self-awareness and confidence (47, 59, 80). We will focus the social domain through the application of diverse group compositions and game arrangements. In this context, we will follow the principles of respect, motivation, and communication while being physically active with other learners (75). Thereupon, we will attempt to create a welcoming and non-judgmental atmosphere to appreciate each participating child in its own individuality (75). Finally, we will acknowledge the behavioral domain as the integrative domain by acting on a child-centered basis, giving the participating children a range of options and free time to choose. In general, the intervention will highly appreciate the individuality of all participants as they “will need to consider which elements are relevant to their own development in order to pursue the activities that will help to develop or maintain physical literacy” (47).

#### 2.3.2. Intervention deliverers

Two coaches will deliver the interventions of this study. One coach (age at the beginning of the study: 26 years) holds a master's degree in pedagogics, a bachelor's degree in pedagogics with physical education as a minor subject. She has a global trainer's license (i.e., without specialization; “Übungsleitungsschein-C Breitensport”) and comprehensive experiences with instructing children in sport contexts. She will be integral part of the academic team and also contribute conceptually to the design of the study. The other coach (age at the beginning of the study: 25 years) holds a bachelor's degree in health management and has also acquired several coaching licenses (e.g., on fitness and yoga for children), gained comprehensive experiences with instructing children in sport contexts, and will be specifically hired for the practical part of the interventions. The two coaches will also individually supervise and conduct the measurements, while gaining support by a second person (i.e., student assistant or instructed teacher from the local school) for the days of assessment. Blinding of the two deliverers cannot be realized as they were specifically employed for the purpose of this study.

#### 2.3.3. Specific training of deliverers

Both coaches will be trained with key elements of the theoretical interpretation of the construct PL prior to the start of implementation. In this context, the coaches will familiarize themselves with the translation of PL in a teaching and learning process (59, 75). The first coach will take a decisive mediator role for the entire training process by (a) being part of the academic team and its scientific discussions of PL, (b) conceptualizing the intervention content and its translation into PL session documents (manual based), and (c) organizing conceptual exchange about PL in general and the 14 sessions in specific. To foster the conceptual rigor in the scope of the implementation, both coaches will undergo external training during the pilot phase by completing a PL development workshop offered by the International Physical Literacy Association (IPLA).

### 2.4. Qualitative instruments

Following the intention to advance the initial intervention program toward the examination of intervention effectiveness in the main study and to gain subjective perspectives of intervention participants, we will integrate a comprehensive qualitative approach within the pilot studies (cycles 1 and 2). In this context, we will focus on the perspective of the intervention deliverers, on the one hand, and the subjective perspective of the participating children, on the other. Moreover, we will install a multiperspective panel to cultivate regular discussions on the delivery and to enable instant revisions of intervention sessions during the intervention phases (formative evaluation). Depending on the results of the qualitative evaluation within the pilot studies, we strive to maintain the qualitative interviews with the children for the main study as well, as they might give relevant insights and experiences beyond the quantitative approach. All interviewees will have to provide informed consent to participation. All children additionally have to return written consent by their legal guardian.

#### 2.4.1. Perspective of intervention deliverers: intervention documentation

To cover the perspective of the intervention deliverers, we will use intervention documentations for each group at the schools and for each session by recording basic information (e.g., start and end time of the intervention, number of participants, name of the school, topic of the session) as well as general feedback toward the session (practicability of games, exercises, the basic structure of the session), potential adaptions and necessary modifications, other incidents (e.g., delays, disputes of children with the coach, issues with the setting) and, finally, the confidence of the deliverer throughout the single session.

#### 2.4.2. Multiperspective panel for intervention discussion and revision

**Figure 2:**
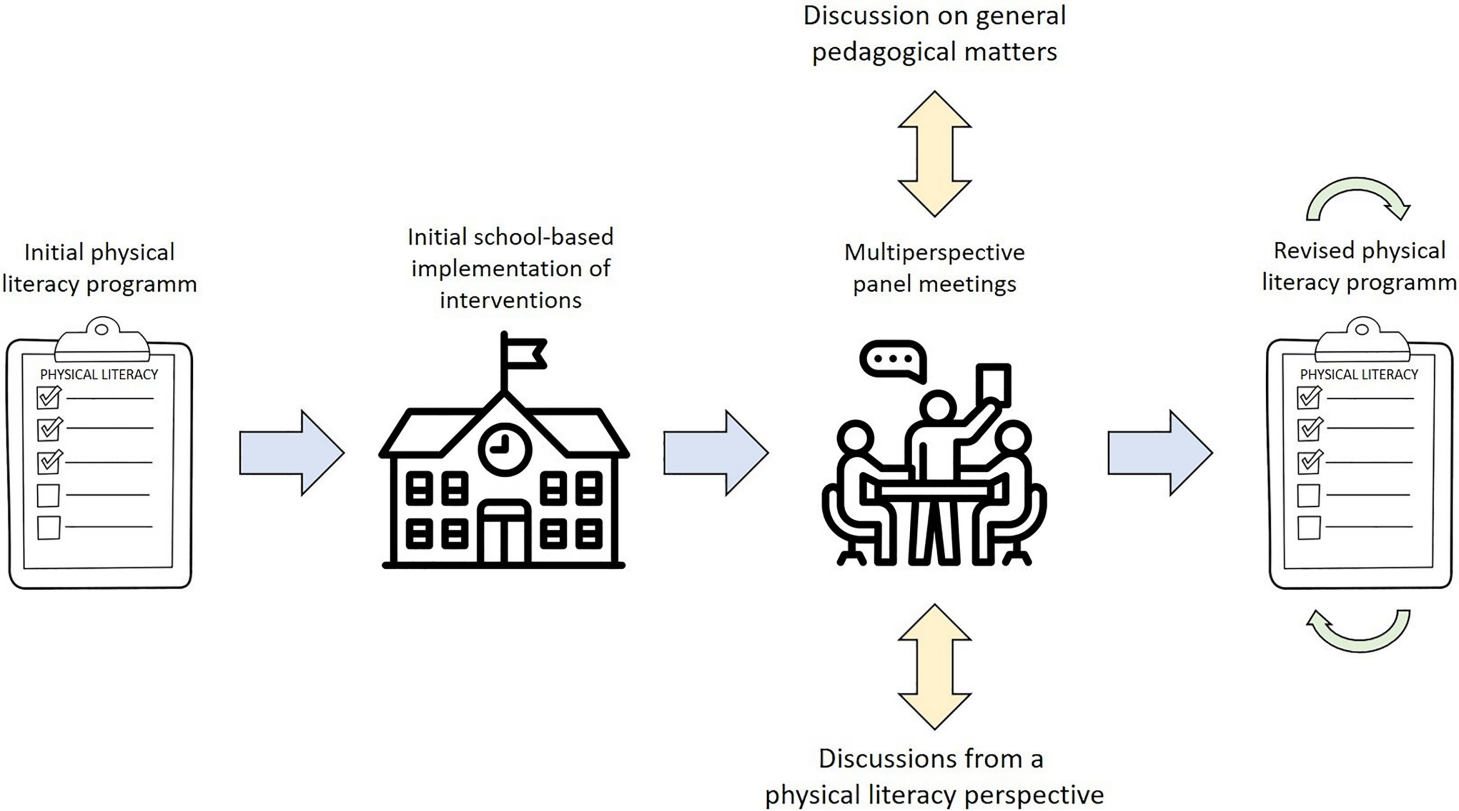
The role of the multiperspective discussion panel for the revision of the intervention (icons were taken from www.flaticon.com).

Moreover, the delivery of the interventions will be accompanied by meetings of an installed panel. This panel can be characterized as: *multiperspective* (as it combines the perspectives of the coaches, of the scientific team, and of the youth welfare service), *multiprofessional* (as it brings together persons from different professional backgrounds and traditions (i.e., general pedagogy, psychology, sport science, health management, social pedagogy) (81), and *transdisciplinary* (as it enables cooperation between researchers and practitioners) (56). These meetings will be held weekly and should, on average, comprise 4–5 participants per session. More specifically, this panel will start with reports of the coaches sharing their concrete experiences from the previous delivery and will lead into open discussion afterwards (see Figure 2). On the one hand, the panel will debate general pedagogical issues (such as the management of children who regularly disrupt the delivery and atmosphere), feedback given by relevant actors of a setting, and restrictions in the intervention settings (e.g., problems in equipment, schedules, room capacities, or in communication with schools and teachers). On the other hand, the panel will also address difficulties with the translation of the PL concept into practice (e.g., challenges with the holistic claim of PL and the associated complexity in delivery). In any case, the defined moderator (rotational procedure) will attempt to maintain a constructive and solution-orientated atmosphere within the session.

#### 2.4.3. Perspective of the participating children: group interviews

As the third part of the qualitative approach, we will interview children after the pilot cycles 1 and 2 (depending on the results, potentially also after the main study) to gain insights into the perspective of participants. More specifically, we will ask the children about their favourite and less favourite topics throughout the interventions (inclusing the underlying reasons), their individual learning achievements, suggestions for the intervention deliverers to improve, and finally their perspective on further participation. We plan to perform 3–5 group interviews, each with 3–6 participating children. Group interviews are defined as semi-structured interviews, focused on a specific topic or theme and guided through the interviewer (54). In this context, we will consider important principles for taking interviews with children, such as a sufficient familiarization with the setting, provision of privacy, the maximum length of the interview, and a trustworthy atmosphere between the interviewer and the participating children (55). Moreover, with a focus on our target group and their level of communicative skills we designed the interview with child-specific incentives and an age-appropriate language (82). The arrangement of a group situation within the interview contributes to overcome potential barriers such as generation differences or dominance relations and is further similar to common school arrangements (82, 83). The group interviews will be led by the second author (LS) and will take place after the quantitative evaluation within selected, interested schools.

### 2.5. Quantitative instruments

In line with the theoretical five-domain structure applied by this study, we will choose a multidimensional assessment strategy to cover the different domains of PL. Due to acceptable measurement characteristics, the second version of the Canadian Assessment of Physical Literacy (CAPL-2) (84, 85), the Canadian Passport for Life (PFL) (86), or the Canadian Physical Literacy Assessment for Youth (PLAY) (87) were taken into account (88), all measuring the cognitive, psychological/affective, and physical domains of PL. As another alternative, the Physical Literacy in Children Questionnaire (PL-C Quest) will be selected as an opportunity for evaluation (89). Based on the Australian understanding of PL, the PL-C Quest is a picture-based self-assessment of the physical, psychological (affective), cognitive, and social domains. Available data from factor analyses indicate a good model fit for boys in the English-speaking setting (90). In detail, the psychometric properties of the procedure are reported as moderate (social domain) to good (physical domain), with model fit being better for boys than for girls. Additionally, the image-based method can be beneficial for children with language or comprehension difficulties. In the present study, we will consistently draw on the “summer items” (e.g., skateboarding instead of skating and safe swimming in the sea instead of safe tobogganing on the slopes) of the available item pool of the PL C Quest. A more detailed overview is given in Table 2.

**Table 2:** A detailed overview of the quantitative assessments in regard to the PL domains.

	Physical domain	Cognitive domain	Psychological (affective) domain	Social domain	Behavioral domain
Pilot study 1 [uncontrolled pre-post design]	PL-C Quest [12 items]	PL-C Quest [7 items] CAPL-2 [5 items]	PL-C Quest [7 items] CAPL-2 [12 items]	PL-C Quest [4 items]	CAPL-2 [1 item]
Pilot study 2 [uncontrolled pre-post design]	PL-C Quest [12 items]	PL-C Quest [7 items] PFL [3 items] PLAYself [2 items]	CAPL-2 [6 items] PL-C Quest[7 items]	PL-C Quest [4 items]	CAPL-2 [1 item]
Main study [controlled design]	DMT 6–18 [8 tests] PL-C Quest [12 items]	Selection based on data from the pilot studies	Selection based on data from the pilot studies	PL-C Quest [4 items]	CAPL-2 [1 item]

CAPL-2, Canadian assessment of physical literacy, second version; DMT, Deutscher Motoriktest; PFL, passport for life; PL-C Quest, physical literacy in children questionnaire.

#### 2.5.1. Physical domain

In the pilot studies, the physical domain will be evaluated using the PL-C Quest (89) and, therefore, a self-assessment procedure. The children are asked to inspect 12 pairs of pictures on different topics (e.g., object manipulation, flexibility, or coordination) and to assign themselves to the picture with the most suitable description. For the main study, we will employ a complementary objective measure of physical competence to map children's development. The assessment will be based on the International Physical Performance Test Profile 6–18 (Deutscher Motorik Test, DMT 6–18) to operationalize this domain (91). This procedure offers the advantage of having age-group and gender-specific norm values.

#### 2.5.2. Cognitive domain

Within the scope of pilot study 1, the assessment of this knowledge and comprehension domain will be collected via seven picture-based items of the PL-C Quest (e.g., rules, anticipation, awareness) (46). Similar as to the physical domain, children assign themselves to the picture with the most suitable description. Additionally, specific knowledge questions of the CAPL-2 are used (e.g., cloze test or multiple-choice questions) (84, 85). Since the scientific community has reported problems with the assessment of the cognitive domain (88, 92, 93), the second pilot study will employ an additional survey for the cognitive domain. For this purpose, we will use the items of the living skills scale of the PFL (86), labelled as *thinking* (exemplary item: “I can use words like run, hop, skip and dodge to explain how I move”), combined with supplementary questions of the PLAYself (exemplary items: “I understand the words that coaches and PE teachers use”) (87). Following the two pilot studies, a decision will be made as to which method is best suited to map the cognitive component.

#### 2.5.3. Psychological (affective) domain

We will record the psychological domain based on seven pairs of images of the PL-C Quest, which illustrate topics like collaboration or dealing with failure. In addition, we will record the affective domain via the CAPL-2, including its subcategories intrinsic motivation, PA competence, predilection, and adequacy (84, 85). Each of these subcategories will be evaluated by a 4-point Likert scale (predilection and adequacy) and a 5-point Likert scale (intrinsic motivation and PA competence), respectively. The answer selection in the Likert scale will be transferred into a score (0.5 or 0.6 points for the lowest PL expression and 2.5 points for the highest PL expression per question), which will result in a total score for this domain. This score will be interpreted by using norm values of the CAPL-2 (85). To subsequently reduce the complexity of the questionnaire, we will survey only the subscales “intrinsic motivation” and “PA competence” in the second pilot study. These two subscales will be selected because they play a particular role in the definition of PL and can be seen as drivers of enduring physical activity (75). Following the comparative evaluation of the two pilot studies, we will make a final decision as to which items are used to survey the psychological domain.

#### 2.5.4. Social domain

The social domain is collected solely via the PL-C Quest (89). The four pairs of images represent the components of relationships, collaboration, ethics, as well as society and culture. It should be emphasized here that the reliability of this subscale was described as only moderate in the initial sample of Barnett and colleagues. Since other evaluation procedures [e.g., the CAPL-2 (85)] do not measure the social domain, this scale is used despite its psychometric weakness and without further scale to validate the measurement.

#### 2.5.5. Behavioral domain

In the pilot study, the participating children will be invited to indicate leisure-time sports and the number of days within a week with a daily PA level above the WHO recommendation of 60 min. The number of days corresponds to the CAPL-2 survey (85).

#### 2.5.6. Further participant characteristics

In addition, we will acquire the following information from the participating children via self-report: gender, age (via birthday date), body mass index (via height and weight), and sport club membership, their potential participation in recreational sports outside school and how the children manage their way to school (actively by foot, bike, scooter or passively by car or public transport).

### 2.6. Sample size

We performed sample size calculations with the software G*Power version 3.1 (94). For the uncontrolled *pilot study* adopting a pre-post character, we expect a minimum effect size of *d_z_* = 0.40 while considering the influence of general development effects. In this case, the minimum sample size to be acquired through one-sided, paired sample *t* tests (statistical power ≥ 80% and one-sided *p* < .05) will be *n* = 41. Assuming a conservative dropout rate of 25% (as usual for this target group, see (14, 95), an initial sample of *n* = 55 children will be recruited. Given an average number of approximately 20 pupils per class, we will include three to four classes for the pilot study.

For the subsequent main study in a control group design, we base a significant effect on time-group interactions of repeated multivariate analyses of variance (MANOVA). Aiming to control for general development effects through the involvement of a control arm, we set a smaller effect size of *d* = 0.30 (corresponding to *f* = 0.15) in this part of the study. Power calculations with an adjusted significance level (five PL outcomes:.05/5) of *p* ≤ .01 resulted in a required sample size of *n* = 134 children across the two study conditions (autoregressive pre-post correlation *r_t_* = 0.50, statistical power ≥ 80%). A total of nine to ten classes (dropout corrected *n* = 179 children) should initially participate in the main study.

### 2.7. Data analysis

#### 2.7.1. Qualitative aspects

All interviews will be voice recorded and transcribed with F4 verbatim. Subsequently, we will subject the entire interview material to qualitative content analysis using MAXQDA 2022. Following Mayring and Fenzl (96), we will combine, explicate, and structure the qualitative data with a main focus on building categories and systems of categories. In accordance with the working model of Mayring, we will focus on reducing, highlighting and verifying representative categories always in line with the entire output material (97). We will first assign interview passages into categories developed within the interview material in an inductive manner and proceed with analyzing and interconnecting the same codes in different sections of the interview (96). To meet qualitative standards of validity and objectivity, we will proceeed with two working steps. To assure intracoder validity, the analysis will be performed by the seond author (LS) in two isolated steps and the results will be converged afterwards (97). Furthermore, a second person (first author) will analyze the entire interview, with the research team subsequently discussing critical interview passages as well as disparities in the results (98).

#### 2.7.2. Quantitative analysis

The *pilot study* using a non-controlled pre-post design will be analyzed with paired sample tests. In this case, the time (pretest vs. posttest) will be treated as the independent variable and the PL domains as the dependent variables. Depending on the support or rejection of normal distribution (based on Kolmogorov Smirnov test), we will compute paired sample *t* tests or Wilcoxon tests for the inferential statistical comparison.

Furthermore, the Transparent Reporting of Evaluation with Non-Randomized Designs (TREND) statement will serve as an overarching framework (99) to guide the design, evaluation, and reporting of the *main study*. We will use a variance analytical design with multivariate character to quantitatively examine the effectiveness of the intervention arm in comparison to the control arm (repeated MANOVA). Statistically, main attention will be paid to the time-group interaction in the repeated MANOVA (within-subjects factor: time; between-subjects factor: group condition). We will determine the magnitude of the intervention effect on the eta square (*η*^2^) coefficient by adhering to the interpretation guidelines as suggested by Cohen (100). Despite the nested structure of data (e.g., classes as clusters), multilevel analysis (i.e., linear mixed methods) cannot be realized owing to the low number of second-order units (*N* ≤ 10 schools). Even robust methods, like the Kenward-Roger correction, require a larger number of clusters for adequate calculations (101). To account for potential clustering at the class level, we will enter the class as covariate within the MANOVA. Missing data in the longitudinal data set will be imputed with the expectation maximization (EM) algorithm (102). Nevertheless, we will withdraw children from the analysis who did not attend (a) the first session with the baseline measurement and (b) at least eleven of the 14 sessions (<75%; i.e., exclusion of those who were missing more than three sessions of the PL intervention).

## 3. Discussion

### 3.1. Physical literacy and the intervention

The PL concept holds promise to address individual's determinants for physically active lifestyles holistically. In this regard, the concept could contribute to the problem that the COVID-19 pandemic has negatively affected levels of and qualifications for adequate PA behaviors. However, although crucial documents in the area of health and policy [e.g., the Global Action Plan on Physical Activity 2018–2030 (15)] have suggested stakeholders to align practices with PL, not all countries acknowledge PL similarly and have facilitated the adoption of this concept by systematically promoting country-specific groundwork (e.g., through theoretical discussions or the development of assessment instruments) (34). This scholarly initiative bases on the assumption that also Germany lacks corresponding experiences with the PL idea (46) and that schools represent the appropriate setting to efficiently reach children along the socioeconomic spectrum.

The present study derives a theory-based PL intervention for children aged 8–11 years and uses the extracurricular time at primary schools to facilitate the child-centered concept outside the traditional compulsory atmosphere. The specificity of this intervention lies in the explicit theory-content links (70), the consideration of integrative principles between the PL domains as well as the consecutive advancements across the two pilot studies. The initial pilot cycles account for potential risks coming up during the preparation of the main study and will be embedded into a mixed-methods design that covers the perspective of both the intervention deliverers and recipients (i.e., children). In the main study, the intervention is finally tested in a controlled design to gather information about its effectiveness.

### 3.2. Limitations

However, the study has the limitation that the logistic situation (i.e., trainer qualification, infrastructure) does not permit to include water-based activities, although aquatic experiences represent an important aspect of PL development (105, 106). Furthermore, the children cannot be randomly assigned to the intervention and control condition. In this context, classes constitute the level of organization, thus making randomizations on the individual level impossible. Unfortunately, we could not realize a cluster-randomized controlled design for two reasons. First, we anticipate a too low overall number of participating classes to ensure a balanced distribution of hypothetical confounders (in the sense of a “robust” randomization). Second, classes were nested in schools and organizational processes within the schools (e.g., the health agent or director of a school), i.e., from third persons outside the academic teams, make it necessary to prescribe the order of classes involved. Nevertheless, the controlled design will allow to give recommendations regarding the potential application of the intervention for children in Germany. In the long term, such evidence-supported interventions can inform national and international practices with a holistic claim toward PA.

### 3.3. Dissemination aspects of this study

In case of a successful main study with results justifying a further use of the PL intervention, the development of a dissemination strategy may come into play. Indeed, the present project has reserved personal, temporal, and financial resources for four months to potentially prepare a dissemination strategy that may flow into a follow-up study. For instance, teachers could be systematically trained for more sustainable, internal delivery in participating schools, potentially paralleled by an entire whole school approach permeating the entire institution (107, 108). A dissemination could also aim at reaching a wider target group by performing a so-called “scale-up” of the intervention (103, 104). Of course, the type of the scale-up depends on the results and their interpretations but also on pragmatic arguments and the development of the scholarly landscape on PL. For instance, a scale-up on the *geographical level* would implicate that also locations outside the city state of Bremen, Germany, could be addressed to reach children in other areas. Adopting the character of a *sectorial scale-up*, the successful intervention could be adapted and used in other settings, such as sport clubs or community-based work. As a further alternative, a scale-up on the *ontogenetic/chronological level* could mean that individuals of another age could benefit from a similar or slightly adapted PL intervention. In any case, dissemination strategies should also test whether the adapted PL intervention then also works in other settings, in other regions, or in individuals of another age.

## Data Availability

The original contributions presented in the study are included in the article/**Supplementary Material**, further inquiries can be directed to the corresponding author.
