# Addendum: Development and evaluation of a school-based physical literacy intervention for children in Germany: protocol of the PLACE study

**DOI:** 10.3389/fspor.2023.1279543

**Published:** 2024-01-31

**Authors:** Katharina Pöppel, Louisa Schmittwilken, Johannes Carl

**Affiliations:** ^1^ Institute of Sport Science, Carl von Ossietzky Universität Oldenburg, Oldenburg, Germany; ^2^ Department of Sport Science and Sport, Friedrich-Alexander University Erlangen-Nürnberg, Erlangen, Germany

**Keywords:** active lifestyle, education, enrichment, exercise, health, physical activity, practice, program

## Abstract

Pilot studies serve to explore the feasibility of assessment instruments and intervention arrangements. As there have not been any validated German physical literacy (PL) assessments for children, we originally included four translated instruments into the pilot phases of the PLACE study, which aimed to test the effectiveness of a theory-based PL intervention for the extracurricular time at primary schools for pupils in Bremen of grades three to four. Unfortunately, the exploratory analyses revealed that all quantitative PL instruments, despite extensive revision throughout the pilot waves, showed insufficient reliability and validity. Against this background, we have to adjust the main phase of the study and deviate meaningfully from our initial study protocol with mixed-methods character [Carl, J., Schmittwilken, L., & Pöppel, K. (2023). Development and evaluation of a school-based physical literacy intervention for children in Germany: protocol of the PLACE study. *Frontiers in Sports and Active Living, 5*, 1155363. doi: 10.3389/fspor.2023.1155363] by dissolving the quantitative part in favor of a qualitative study with multiple data sources (multiperspective discussion panel, session protocols, group interviews). We want to transparently communicate this re-arrangement prior to the main phase.

An Addendum on Development and Evaluation of a School-Based Physical Literacy Intervention for Children in Germany: Protocol of the PLACE Study By Johannes Carl, Louisa Schmittwilken and Katharina Pöppel (2023). Front. Sports Act. Living Sec. Physical Education and Pedagogy. 5: doi: 10.3389/fspor.2023.1155363.

## 1 Physical literacy and the PLACE study

The concept of physical literacy (PL) can be characterized as a holistic approach, that considers different determinants for a physically, active lifestyle throughout the life course (1, 2). Based on the assumption that PL has the potential to mitigate the declines in physical activity (PA) after the COVID-19 pandemic (3), our research team has developed the PLACE study for extracurricular education of children in grades three and four (age: 8–11 years) in schools in the city state of Bremen, Germany. The intervention comprises 12 different sessions plus two charting sessions while demonstrating explicit links between the theoretical PL components in accordance with the Australian framework (4) and the intervention content. Given the conceptual, psychometric, and interventional groundwork to be done for PL in the German context (5), the study has drawn on a multiphase design providing two pilot cycles and one subsequent main study. In correspondence with a mixed-methods approach, we have defined a *qualitative evaluation* (a multiperspective discussion panel; intervention documentation by the deliverers; group interviews with the participating children) and a *quantitative evaluation* with different instruments each specifying an operationalization of the different PL domains. In line with recommendations favoring an a-priori definition of the study design, of intervention components, and of evaluation instruments (6), we have recently published a study protocol that describes the procedure of our intervention study (7). However, the experiences and findings of the two pilot cycles induce us to deviate significantly from the original evaluation approach for ensuring the validity of the findings of the upcoming main study. Therefore, the goal of this short addendum is to transparently describe the modified evaluation approach of the PLACE study, which follows the goal to evaluate the effectiveness of the German PL intervention with a control group design. We again highlight that this addendum is set up in the run-up to the start of the main phase.

## 2 Background to the original assessment

During the evaluation, we faced challenges that made adaptions for the main study necessary. The following paragraphs underpin the adjustments to the study protocol. Initially we integrated a series of internationally published and accepted PL instruments into the pilot studies with the intention to also test their validity in German language, as there were, to the best of our knowledge, no published German translations available: the Canadian Assessment of Physical Literacy (CAPL-2) (8), the Passport for Life (PFL) (9), the Canadian Physical Literacy Assessment for Youth (PLAY) (10, 11), and the Physical Literacy in Children Questionnaire (PL-C Quest) (12, 13). The inclusion of the assessments was based on psychometric information retrieved from the original versions in English language (14, 15). In the international context, providing a PL-specific operationalization that includes all domains is still challenging and a point of discussion (16). This also includes the suitability of the measurements for mapping PL changes in children.

## 3 Assessment issues during the pilot studies

As part of pilot study 1, we applied the PL-C Quest for the physical domain, the PL C-Quest and the CAPL-2 for the cognitive domain, the PL C-Quest and the CAPL-2 for the psychological domain, the PL C-Quest for the social domain, and the CAPL-2 for the behavioral domain. In a first step, we conducted exploratory item analyses, which finally unfolded several problems: Except for one item, all items showed right skewed item-distributions ranging from −2.07 to −0.6 (*M* = −1.17, *SD* = .48). Furthermore, the item-total correlations ranged from −.06 to .68. Solely ten out of 30 items showed values above the recommended item-total correlation of .50 (17). Finally, we faced high item difficulty values ranging from .46 to .94 (*M* = .77, *SD* = 0.92). The psychometric properties of the individual subscales indicated that the applied measurements cannot differentiate between the measurement time points. On the qualitative level, we received the feedback that the children showed difficulties understanding the processing of the items during the image-based questionnaire (PL C-Quest) and the text questions (CAPL-2). Additionally, some children expressed frustration. Despite the PL coaches’ supervision of the questionnaire and support, it could not be ensured that the children had answered the questions correctly. The processing time varied considerably among the children and took, on average, longer than expected.

In pilot study 2, we applied the PL-C Quest for the physical domain, the PL C-Quest and the PFL as well as PLAYself for the cognitive domain, the PL C-Quest and the CAPL-2 for the psychological domain, the PL C-Quest for the social domain, and the CAPL-2 for the behavioral domain. Based on our experiences from pilot cycle 1, we undertook slight adjustments to the items, including a simplification of the formulations of the PL C-Quest, and also tried out another media format (tablet). Nevertheless, we registered similar problems (as in pilot study 1) in terms of skewness (*M* = −.90, *SD* = .52, range: −1.93 to 0.93) with 25 out of 30 items significantly deviating significantly from normal distribution, item-total correlations (range: .08 to .55) with solely four out of 30 items being located above the recommended value of .50 (17), and on average high item difficulties (*M* = .73, *SD* = .12). These empirical values were again paralleled by negative reports from the participating children and the supervising instructors. In summary, the project team had to determine before the post-test of pilot study 2 that no included assessment (or at least combination of assessments) had the quality to validly and reliably differentiate between the different PL domains. Against this backdrop, we established that the initial evaluation strategy can't be maintained both scientifically and pragmatically.

Based on these experiences, we decided to re-arrange the strategy within our mixed-methods design as documented in the study protocol. We decided to further adhere to the control group design but to extend the qualitative procedure and instead to place stronger emphasis on comparisons between the PL approach and regular physical education in the main study. As a quantitative supplement, we will use a non-standardized, one-page paper-based questionnaire in the main study, which was explored in the post-test of pilot study 2. It consists of a selection of subscales and items covering the four PL domains and records the children's responses on a smiley-based 4-point Likert scale. The physical domain was represented by three items of the CAPL-2 subscale Physical Activity Competence, the psychological domain was operationalized using the three items of the pleasure scale of a questionnaire for measuring student's enjoyment in physical education classes (FEFS-J) (18), the cognitive domain consisted of three items from the subscale cognitive of the PFL (9), the social domain was based on items of the assessment of social competencies in children and adolescents (19), and the final item measuring the behavioral domain was based on the PFL (9). The exploratory data analyses for the post-test of pilot study 2 also showed right-skewed distributions, but comparably better item-total correlations ranging from .40 to .71 with nine out of 12 items showing values above .50. Nevertheless, the development status of this instrument does not justify its employment in this study as a primary outcome, as the number of items and validity of the questionnaire have not been sufficiently established. In line with the definition of PL, the children's self-assessment was given primary importance for the main study. Accordingly, the project group also decided to refrain from carrying out objective motor tests.

## 4 Qualitative approach

The initial findings made it necessary to dissolve from the quantitative evaluation, instead to prioritize the qualitative approach. As reported in the initial study protocol (7), we will describe the multi-step intervention development through intervention documentation (via structured protocols), a multiperspective discussion panel, and group interviews with the children participating in the intervention. For the main study, we will maintain these multiple data sources for gathering information relevant for the evaluation. However, we will increase the number of interviews for this stage of the study design (approx. 4–6 interviews), attempting to cover a balanced number of classes between the two supervising coaches. The interview guide will base on categories identified in the qualitative approach of pilot phases 1 and 2. In this context, we will also inductively (emerging from the interview material with children, complemented through the topics of the expert panel) and deductively (from basic assumptions of PL literature) generate categories that allow a systematic comparison between the present PL intervention (conducted in the extracurricular time of primary schools) and regular physical education. Lastly, the coaches will add an informal poster rating with physical, cognitive, psychological, and social elements the delivery as part of the final charting session to acquire group-based information about the PL domains.

## 5 Discussion

Experiences from implementing the PLACE study within pilot phases 1 and 2 made it necessary to adjust the mixed-methods evaluation approach. Striving for a valid PL evaluation via self-report, we had to react to difficulties in the pilot studies. The simplifications mirror children's reading and comprehension capacities, which in the city state of Bremen often rank among the weakest in Germany (20).

## Ethics statement

The studies involving humans were approved by Carl von Ossietzky Universität Oldenburg, Germany, Local Ethics Committee. The studies were conducted in accordance with the local legislation and institutional requirements. Written informed consent for participation in this study was provided by the participants’ legal guardians/next of kin.
